# Jejunal Diverticulosis Complicated by Diverticulitis and Small Bowel Obstruction

**DOI:** 10.7759/cureus.8347

**Published:** 2020-05-28

**Authors:** Yousef Elfanagely, Chung Sang Tse, Priyanka Patil, Stephanie Lueckel

**Affiliations:** 1 Internal Medicine, Warren Alpert Medical School of Brown University, Providence, USA; 2 Gastroenterology, Warren Alpert Medical School of Brown University, Providence, USA; 3 Pathology, Warren Alpert Medical School of Brown University, Providence, USA; 4 Trauma and Surgical Critical Care, Warren Alpert Medical School of Brown University, Providence, USA

**Keywords:** diverticular disease, small bowel diverticulosis, diverticulitis, small bowel obstruction, jejunal diverticulosis

## Abstract

Diverticular disease is common in the Western population and can cause considerable morbidity. The prevalence of colonic diverticulosis reaches 60% by the age of 60 years. Small bowel diverticulosis is much rarer and, when present, most commonly occurs in the duodenum. We herein report an elderly woman with jejunal diverticulosis complicated by diverticulitis and small bowel obstruction, who subsequently underwent small bowel resection and primary anastomosis. As demonstrated by this case, jejunal diverticulitis can cause serious complications and given the possibility of recurrence and serious complications, surgical options should be discussed early in the course of medical care.

## Introduction

Jejunal diverticulosis is a rare condition with an incidence of 0.06% to 5% based on radiographic and autopsy studies [1]. Patients are usually asymptomatic and diagnosed incidentally on radiographic imaging. In a minority of patients, jejunal diverticulosis can manifest with clinical symptoms and develop serious complications, including diverticulitis, hemorrhage, intussusception, small bowel obstruction, or perforation [2]. We herein present a case of an elderly female who presented with acute abdominal pain and found to have jejunal diverticulitis that was complicated by small bowel obstruction with resultant surgical resection. 

## Case presentation

A 79-year-old woman with a history of diabetes and hypertension presented to the emergency department (ED) with severe abdominal pain. Vital signs were notable for hypertension (blood pressure 145/54 mmHg), but otherwise within normal limits and afebrile. Abdominal exam was significant for positive bowel sounds, a soft and non-distended abdomen, and epigastric and left upper abdominal tenderness, with no guarding or rigidity. The remainder of the physical exam was unremarkable. Laboratory testing was notable for leukocytosis with white blood cell (WBC) of 19.8 x 10^9^/L (normal 3.5-11.0 x 10^9^/L) and acute renal failure with creatinine 1.45 mg/dL (baseline creatinine 0.9) (normal 0.44-1.03 mg/dL) (Table 1).

**Table 1:** Reference range of lab values

Lab values	Reference range
White blood cell (WBC)	3.5-11.0 x 10^9^/L
Creatinine	0.44-1.03 mg/dL

**Figure 1:**
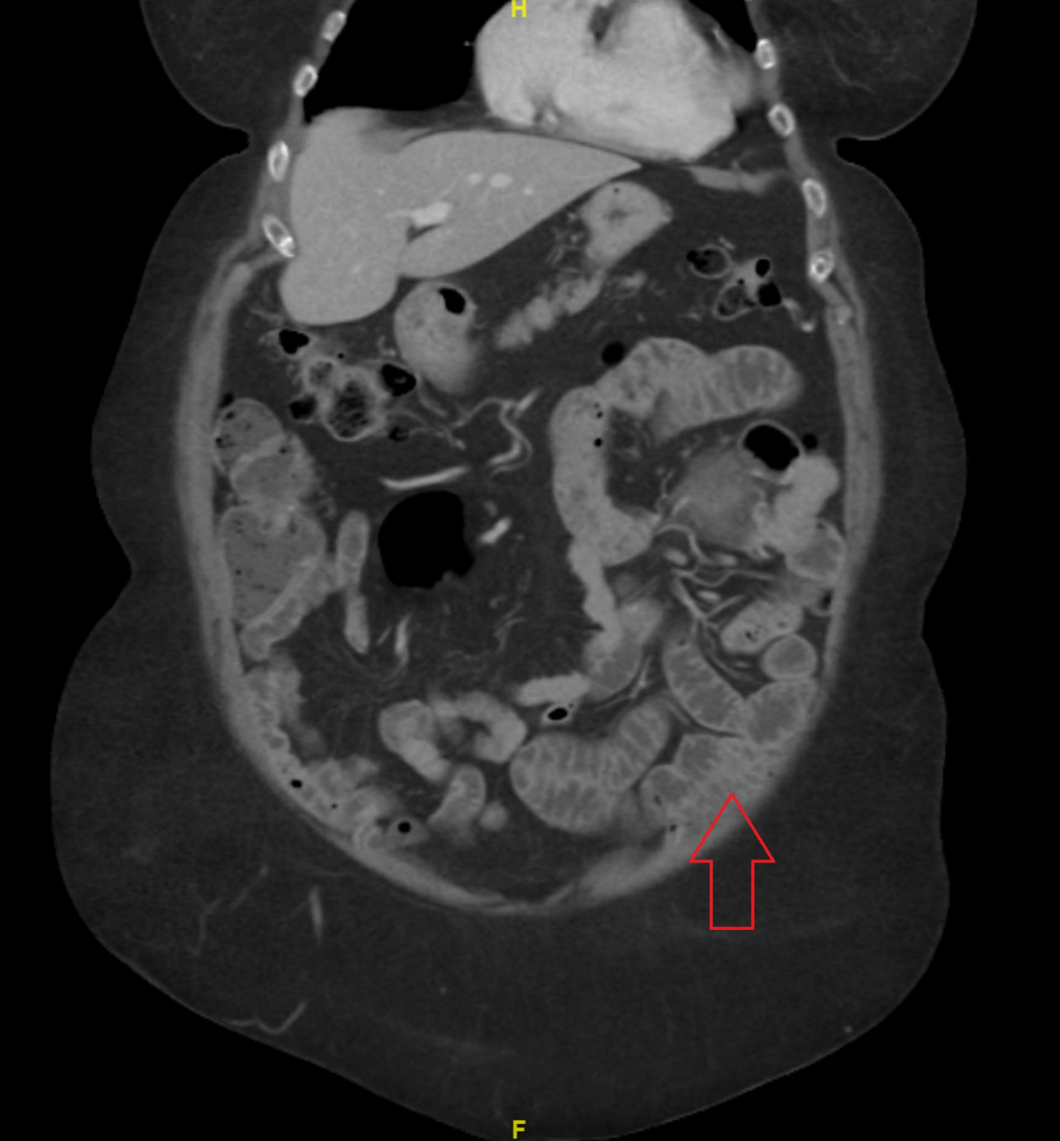
Jejunal diverticulum with surrounding small amount of fluid and inflammatory change (adnominal CT with contrast – coronal view)

Abdominal computed tomography (CT) with contrast showed a jejunal diverticulum with surrounding small amount of fluid and inflammatory change, consistent with acute uncomplicated jejunal diverticulitis (Figure 1). She was treated with bowel rest, intravenous fluids, and antibiotics (ciprofloxacin HCl 500 mg BID and metronidazole 500 mg q8h for a 15-day course). Upon resolution of her abdominal pain and tolerance of an oral diet on hospital day 9, she was discharged home. 

**Figure 2:**
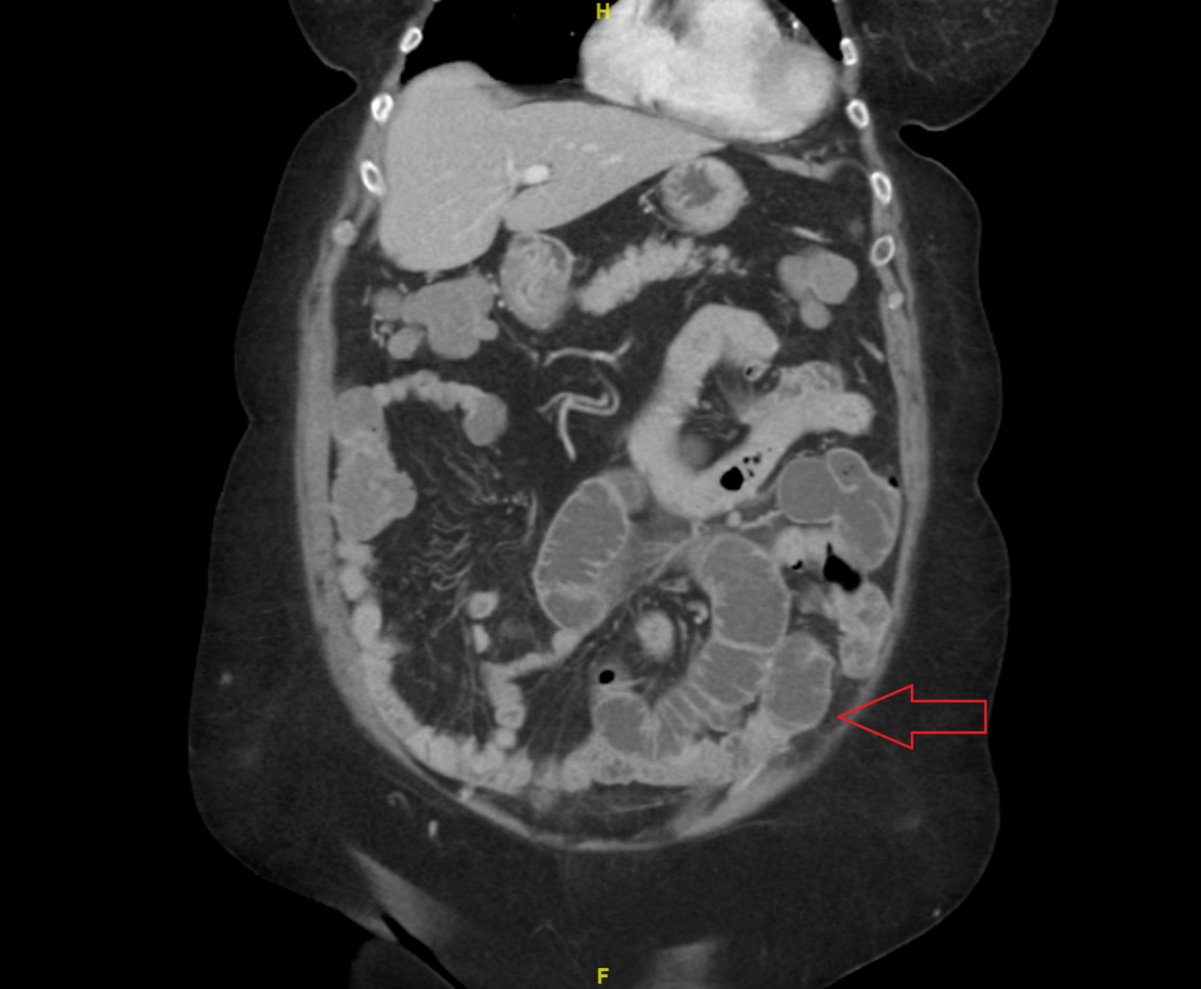
Dilatation of multiple distal loops of the small bowel in the left lower quadrant of the abdomen (abdominal CT with contrast – coronal view)

Six days after hospital discharge, she represented to the ED with generalized, non-radiating abdominal pain, abdominal distention, obstipation, and constipation for seven days. Vital signs were significant for hypertension (blood pressure 147/94 mmHg), but otherwise afebrile and within normal limits. Physical exam was pertinent for abdominal distension and generalized tenderness without abdominal rebound or guarding. Laboratory workup was notable for leukocytosis with WBC 13.9 x 10^9^/L. Abdominal CT with contrast demonstrated dilatation of multiple distal loops of small bowel in the left lower quadrant of the abdomen, suggestive of small bowel obstruction involving the jejunum (Figure 2). 

**Figure 3:**
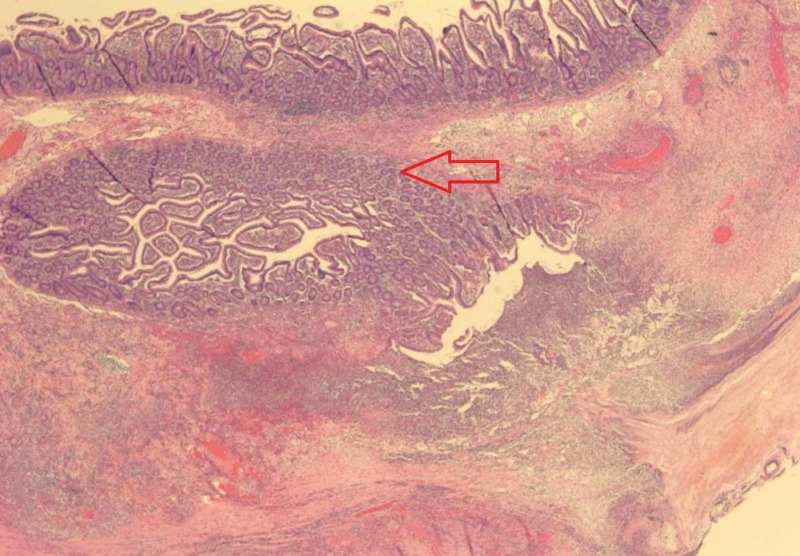
Small intestinal wall with diverticulitis and peridiverticular abscess (hematoxylin and eosin; original magnification: 20X)

**Figure 4:**
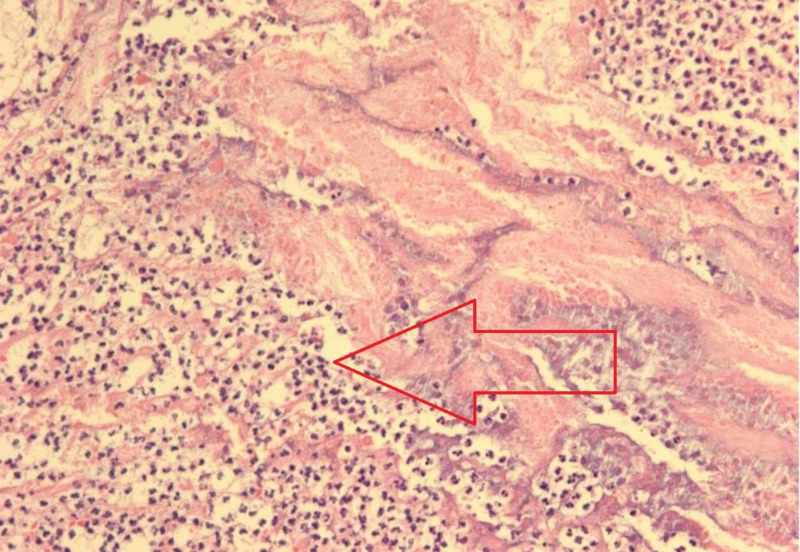
Area of focal necrosis of small intestinal wall (hematoxylin and eosin; original magnification: 100X)

The patient was admitted to the surgical team and she was initially managed medically with bowel rest, intravenous fluids, piperacillin-tazobactam 2.25 gram/50 mL q6h, and bowel decompression with nasogastric (NG) tube placement. One day after her admission, the patient had a return of normal bowel function (flatus and bowel movement). However, given her subacute recurrence of jejunal diverticulitis complicated by small bowel obstruction, she was advised to have a jejunal resection to prevent future episodes of diverticulitis and/or small bowel obstruction. Exploratory laparotomy with small bowel resection and primary anastomosis was performed. During the operation, multiple diverticulae in the jejunum were noted. Histopathology was notable for small intestinal wall with diverticulitis and peridiverticular abscess (Figure 3) and focal necrosis of small intestinal wall (Figure 4). Postoperatively, she had resumption of bowel movements and tolerated an oral diet prior to discharge on postoperative day 5. On outpatient follow-up two weeks later, she was clinically well: tolerating oral diet, having regular bowel movements, no abdominal pain, and no evidence of drainage or infection surrounding the incision.

## Discussion

Intestinal diverticulosis is defined by the presence of diverticula (sac-like protrusions) that can occur in any part of the gastrointestinal tract [3]. Diverticula can be classified by anatomic location and etiology, either congenital or acquired. The most common location of diverticula is the colon [4]. Small bowel diverticula are rare and often found incidentally. In the small bowel, the duodenum is the most common site of diverticulosis, followed by the jejunum and ileum [5,6]. Meckel’s diverticulum is the most common congenital malformation of the gastrointestinal tract, occurring in 2% of the population. It is typically located in the distal ileum 2 feet from the ileocecal valve [7,8]. 

Jejunal diverticulosis has an incidence of 0.06% to 5% [1]. The risk of jejunal diverticulosis increases with age, with the reported median age of 72 to 82 years [9]. Patients with jejunal diverticulosis are often asymptomatic, though up to 40% of patients with jejunal diverticulosis have abdominal pain, changes in bowel habits, and malabsorption [2]. Complications of jejunal diverticulosis, estimated to occur in 10% of patients with jejunal diverticulosis, are serious, and potentially life-threatening, including jejunal diverticulitis, hemorrhage, obstruction, and perforation [1,2,5].

We presented a unique case of jejunal diverticulosis with two sequential complications: jejunal diverticulitis and small bowel obstruction. While the American Society of Colon and Rectal Surgeons recommends non-operative treatment for uncomplicated sigmoid diverticulitis and consideration of elective colectomy after the patient recovers from an episode of complicated diverticulitis (including perforation, abscess, fistula, obstruction, or stricture), there is no consensus for small bowel diverticulitis management, particularly in patients with chronic symptoms [2,10,11]. Some providers believe patients with chronic symptoms can be treated conservatively if the inflammation is mild [11-13]. Others recommend a more aggressive surgical approach given the potential for perforation and development of abscesses [12-14]. As demonstrated by our own case, elective surgical resection after complicated jejunal diverticulitis should be considered if recurrence is likely and symptoms persist. 

## Conclusions

Jejunal diverticulosis is a rare condition that is often asymptomatic. This case presented two sequential complications of jejunal diverticulosis: jejunal diverticulitis and small bowel obstruction. Surgical resection was required which demonstrates the importance of early discussions of elective surgical intervention in patients with jejunal diverticulosis whose symptoms persist. 
